# Hepatic Arterial Infusion Chemotherapy with Oxaliplatin Plus Raltitrexed as an Alternative Option in Advanced Hepatocellular Carcinoma Patients with Failure of, or Unsuitability for, Transarterial Chemoembolization

**DOI:** 10.3390/medicina58101343

**Published:** 2022-09-24

**Authors:** Yanfang Wu, Susu Zheng, Zhenzhen Zhang, Guobin Chen, Xiaochun Chen, Tanghui Zheng, Xinkun Guo, Hong Chen, Meixia Wang, Xiaoying Xie, Boheng Zhang

**Affiliations:** 1 Department of Hepatic Oncology, Xiamen Clinical Research Center for Cancer Therapy, Zhongshan Hospital, Fudan University (Xiamen Branch), Xiamen 361015, China; 2 Key Laboratory for Carcinogenesis and Cancer Invasion of Chinese Ministry of Education, The Liver Cancer Institute, Zhongshan Hospital, Shanghai Medical School, Fudan University, Shanghai 200032, China; 3 Center for Evidence-Based Medicine, Shanghai Medical School, Fudan University, Shanghai 200032, China

**Keywords:** hepatic arterial infusion chemotherapy, hepatocellular carcinoma, transarterial chemoembolization, treatment failure, unsuitability

## Abstract

*Background and Objectives*: To assess the efficacy and safety of hepatic arterial infusion chemotherapy (HAIC) with oxaliplatin plus raltitrexed (HAICROX) as an alternative treatment option for advanced hepatocellular carcinoma (HCC) patients who are ineligible for, or failed, the transarterial chemoembolization (TACE) treatment. *Materials and Methods*: From July 2020 to November 2021, a total of 35 HCC patients were enrolled and received HAIC with oxaliplatin plus raltitrexed. The overall survival (OS) and time to progression (TTP) were primary and secondary endpoints, respectively. The tumor response was assessed by the modified response evaluation criteria in solid tumors (mRECIST), and the adverse events were investigated using the common terminology criteria for adverse events version 5.0 (CTCAE 5.0). *Results*: The median OS and TTP were 10 months (95% confidence interval (CI): 5.5–14.6) and 3.5 months (95% CI: 2.3–4.7), respectively. By means of multivariate analysis, anti-programmed cell death protein 1 (anti-PD-1) immunotherapy was found to be an independent prognostic factor for better survival. No patients experienced toxicity-related death. Thrombocytopenia, alanine aminotransferase (ALT), and aspartate aminotransferase (AST) elevation were the most common toxicities. No grade 3 or higher adverse events related to HAICROX were observed. *Conclusion*: HAICROX showed valuable efficacy and tolerable toxicity in advanced HCC patients who progressed on TACE or were ineligible for TACE. HAICROX is a promising treatment for advanced-stage HCC patients with TACE failure or ineligibility.

## 1. Introduction

Hepatocellular carcinoma (HCC) is one of the most common malignancies and the fourth leading cause of cancer-related death worldwide [1]. HCC is also the third most common cause of cancer-related deaths in China [2]. As HCC is often asymptomatic, most patients are already in the intermediate and advanced stages when first diagnosed. The prognosis is dismal, with a median untreated survival time of 7–9 months [3]. Although tyrosine kinase inhibitors (TKIs) and immune checkpoint inhibitors are regarded as preferred treatments [4], TACE is currently recognized as the most commonly used method for the non-surgical treatment of advanced liver cancer [5,6,7]. However, TACE is not suitable for patients with diffuse HCC, an arterioportal/arteriovenous shunt, or main portal vein tumor thrombosis [5], who are considered as TACE-ineligible. Meanwhile, some patients are classified as TACE-refractory, defined as having refractoriness to TACE after more than two TACE procedures within 6 months [8]. However, there are currently no consistent conclusions on later-line treatments with TACE-refractory or -ineligible patients.

Hepatic arterial infusion chemotherapy (HAIC) is a method of local treatment with a high drug concentration in the liver and few systemic adverse reactions. Several studies have shown that the combination therapy of sorafenib (SORA) plus FOLFOX4-HAIC (the infusion of 5-fluorouracil, leucovorin, and oxaliplatin) showed better survival benefits compared with SORA alone [9,10,11]. In addition, Hsu found that HAIC with FOLFOX4 also showed acceptable outcomes in advanced HCC patients who failed, or were unsuitable for, TACE; the overall survival (OS) and progression-free survival (PFS) were 9.0 months (95% CI: 7.6–10.4) and 3.7 months (95% CI: 3.1–4.3), respectively.

However, the transarterial infusion of 5-fluorouracil needs approximately 44–48 h because 5-fluorouracil is a time-dependent chemotherapy drug. Prolonged arterial infusion is inconvenient and increases the risk of intrahepatic catheter thrombosis and displacement [12]. Conversely, raltitrexed is another thymidylate synthase inhibitor with a longer half-life than 5-Fu, reaching 198 h, and can exert a stable anti-tumor effect over a long period of time [13]. It was regarded as a better candidate for HAIC in HCC. A retrospective study showed that hepatic arterial infusion with a low dose of raltitrexed plus oxaliplatin (HAICROX) was effective in patients with advanced HCC with MPVTT; the median survival time was 8.7 months [14]. A phase II prospective study performed HAICROX in patients with intermediate and advanced-stage HCC; the ORR was 18 (51.4%) out of 35 patients, the DCR was 31 (79.5%) out of 35 patients, the median TTP was 6.7 months (95% CI: 4.6–8.8), and no treatment-related grade 3 or 4 toxicities or deaths were found [15]. However, the efficacy and safety of HAICROX treatment for TACE-refractory or -ineligible advanced HCC patients remain unknown.

Here, we carried out this study to investigate the efficacy and safety of HAICROX for advanced HCC patients with TACE failure or ineligibility.

## 2. Materials and Methods

### 2.1. Patients

The inclusion criteria were: (1) pathologically or radiologically (contrast-enhanced magnetic resonance imaging (MRI) or computed tomography (CT)) confirmed advanced HCC based on the American Association for the Study of Liver Disease criteria. (2) TACE refractoriness/failure, defined as disease progression after more than two TACE sessions (The Japan Society of Hepatology (JSH) defines refractoriness to TACE as a failure to control target lesions or the appearance of new lesions, even after two or more consecutive TACE sessions). TACE ineligibility was defined as diffuse HCC, with major portal vein cancer metastasis, a severe arterioportal/arteriovenous shunt on angiography, tumor thrombosis in the inferior vena cava or right atrium, having received TACE treatment at least once. (3) Patients had at least one measurable lesion. (4) Patients had a Child–Pugh score of ≤6 and an Eastern Cooperative Oncology Group (ECOG) performance status of ≤ 1.

The exclusion criteria were: uncontrollable infection; hepatic encephalopathy; gastrointestinal bleeding; refractory ascites; serum bilirubin levels > 3.0 mg/dL; ALT and AST more than 3 times the upper normal limit; albumin levels < 2.5 g/dL; platelet count < 50 × 109/L; and serum creatinine levels > 1.5 mg/dL. This study was approved by the ethical review board of our institution. Informed consent was waived for all patients. Between July 2020 and November 2021, a total of 35 patients with advanced-stage HCC, confirmed as being TACE-refractory or -ineligible and treated with HAICROX, were included. The demographic, clinical, and survival information was extracted from electronic medical records.

### 2.2. HAIC Procedures

For the HAIC with the oxaliplatin plus raltitrexed procedure, the Seldinger technique was performed through the femoral artery or the radial artery, and a catheter and a coaxial microcatheter were inserted into the feeding hepatic artery. The following regimens of ROX were administered: oxaliplatin (67–75 mg/m^2^ continuous infusion for 4 h) and raltitrexed (2 mg/m^2^ continuous infusion for 1 h). After priming chemotherapy, we removed the catheter and microcatheter, performed the treatment every 3–4 weeks, and then discontinued the treatment, as previously defined [15].

### 2.3. Assessment and Follow-Up

Liver function tests; routine blood tests; and determinations of cytokine, alpha-fetoprotein (AFP), and prothrombin (induced by vitamin K absence-II (PIVKA-II)) levels were performed before every treatment period, and contrast-enhanced CT or MRI of the upper abdomen was performed every 2–3 cycles of HAIC therapy to assess the treatment outcome, according to the modified response evaluation criteria in solid tumors (mRECIST). A further examination was performed if patients had suspected extrahepatic spread. As in a previous study [16], the albumin–bilirubin (ALBI) score was calculated using the following formula: ALBI score = (0.66 × log10 total bilirubin (µmol/L)) + (−0.085 × serum albumin (g/L)). The last follow-up date was 13 March 2022.

The ORR was defined as the number of patients who achieved either a partial response (PR) or a complete response (CR). Additionally, the disease control (DCR) was calculated as the sum of the CR, PR, and stable disease (SD). The primary endpoint of our study was OS, and the secondary endpoint was TTP. In this study, the OS was defined as the period from the initiation of HAIC treatment to death or the last known follow-up, and the TTP was defined as the time from the start of HAIC therapy to disease progression (a radiological or clinical evaluation by mRECIST).

The grade of toxicity was recorded and graded according to the National Cancer Institute’s common terminology criteria for adverse events (CTCAE, version 5.0).

### 2.4. Statistical Analysis

For baseline characteristics, the categorical variables are described as frequencies and percentages, while continuous variables are presented as the mean ± standard deviation (SD). The Kaplan–Meier method was used to estimate the TTP and OS. Univariate analyses were performed using the log-rank test. For the univariate analysis results, variables with a *p* < 0.05 were entered into the multivariate analysis. The multivariate Cox model was applied to identify independent risk factors. All statistical tests were 2-sided, and *p* < 0.05 was considered statistically significant. SPSS software (SPSS version 22.0; SPSS, Chicago, IL, USA) was used to perform the statistical analyses.

## 3. Results

### 3.1. Patients Characteristics

In total, between July 2020 and November 2021, 35 HCC patients with either TACE unsuitability or refractoriness received HAICROX treatment and were enrolled in this study. The baseline demographics and characteristics of enrolled patients are shown in Table 1.

All patients received TKI treatments; among them, 30 patients were refractory to TACE and 5 patients were ineligible for TACE because of major vascular tumor thrombosis. The average age of patients was 53 ± 11.7 years, and the median follow-up period was 10 months (range: 2–17.2). Most patients with an advanced stage of HCC were male (33/35, 94.3%) and had hepatitis B (30/35, 85.7%), a small number of patients had hepatitis C (2/35, 5.7%), and just one patient (2.9%) had an HBV and HCV co-infection. Most patients (34/35, 97.1%) belonged to Child–Pugh class A, and 21 (60%) and 14 (40%) patients had liver functions of ALBI grade 1 and grade 2, respectively. Twenty (57.1%) patients had ECOG 1. Twenty-five (86.7%) patients had more than three tumors. In total, 21 (60%) patients had portal vein thrombosis (PVTT), while 7 (20%) patients had tumor thrombosis in the inferior vena cava. Nineteen (54.3%) patients had extrahepatic metastasis, including four (11.4%) patients with pulmonary metastasis, four (11.4%) patients with bone metastasis, seven (20%) patients with lymph node metastasis, three (8.6%) patients with abdominal metastasis, and one (2.9%) patient with gallbladder invasion. The median number of previous TACE sessions was 2.7 ± 2.3. Meanwhile, 13 (37.1%) patients had received more than the second line of TKI treatment and 22 (62.9%) patients had received anti-PD-1 immunotherapy.

### 3.2. Efficacy and Safety

#### 3.2.1. Tumor Response

The mean number of HAIC treatments was 2.3 (range: 1–5). At 2 months after HAIC treatment, 4 (11.4%) patients had a PR, 16 (45.7%) patients had an SD and 15 (42.9%) patients had a PD. Accordingly, the ORR was 11.4% and the DCR was 57.1%. Table 2 lists the treatment outcome details.

#### 3.2.2. Survival Outcome

**Figure 1:**
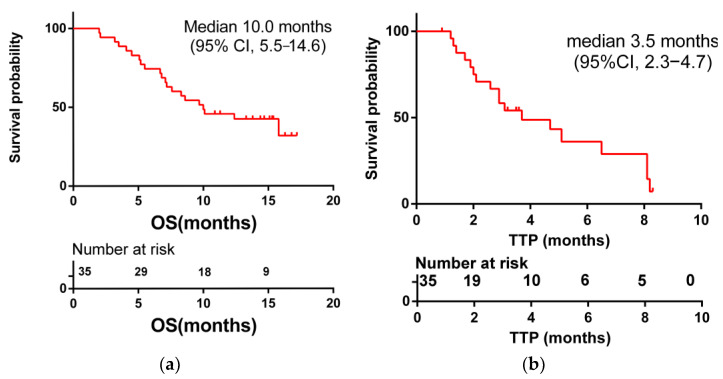
Kaplan–Meier curves for overall survival (OS) (**a**) and time to progression (TTP) (**b**) in 35 patients undergoing HAIC with oxaliplatin plus raltitrexed (HAICROX).

**Figure 2:**
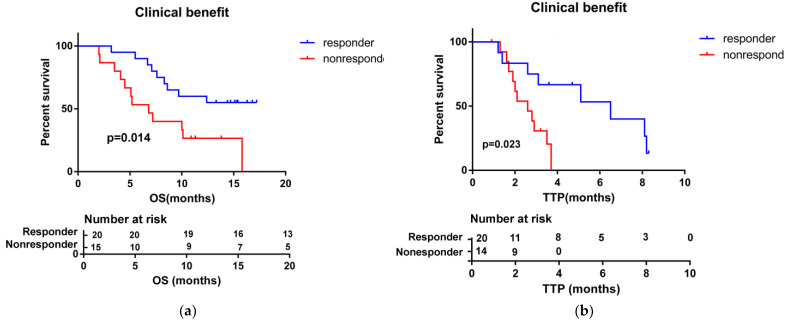
Kaplan–Meier curves for overall survival (OS) (**a**) and TTP (**b**) for patients with clinical benefits (CR + PR + SD) and without clinical benefits (PD) in HCC patients treated with HAICROX, who experienced TACE treatment failure or unsuitability.

**Figure 3:**
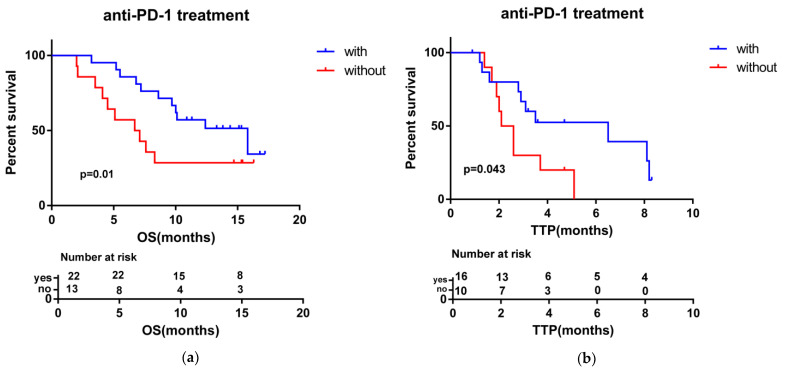
Kaplan–Meier curves for OS (**a**) and TTP (**b**) in patients, stratified by treatment with or without anti-PD-1 immunotherapy.

In total, 21 out of 35 (60%) patients had died at the end of follow-up (March 2022). The mean follow-up time was 10 months (range: 2–17.2). The median OS and TTP were 10 months (95% CI: 5.5–14.6) and 3.5 months (95% CI: 2.3–4.7), respectively (Figure 1). The median OS for patients who achieved a response (PR) to HAIC treatment was not reached, and the median OS for patients with SD and PD (non-respondents) was 8.6 months (95% CI: 5.5–11.8). Patients who achieved clinical benefits (CR + PR + SD) showed a longer OS and TTP compared with people without clinical benefits (median OS, not reached vs. 6.8 months, *p* = 0.014; median TTP, 6.5 months vs. 2.6 months, *p* = 0.023) (Figure 2). These results show that achieving a clinical benefit could predict the patients’ survival. In addition, patients with anti-PD-1 combination therapy showed better OS and TTP than without anti-PD-1 immunotherapy (median OS, 15.8 months (95% CI 7.4–24.2) vs. 6.7 months (95% CI 3.7–9.8), *p* = 0.01; median TTP, 6.5 months (95% CI 1.6–11.4) vs. 2.1 months (95% CI 1.1–3.0), *p* = 0.043) (Figure 3).

#### 3.2.3. Safety and Toxicity

No treatment-related deaths occurred, and no patients experienced grade 3 or 4 toxicities. The most common toxicities associated with HAIC included AST and ALT elevation (both 34.3%), thrombocytopenia (17.1%), bilirubin elevation (8.6%), general weakness (8.6%), dyspepsia/anorexia (2.9%), nausea/vomiting (2.9%), gastrointestinal (GI) bleeding (2.9%), hyponatremia (2.9%), ascites aggravation (2.9%), and hepatic encephalopathy (2.9%). HAIC-related toxicities are detailed in Table 3.

### 3.3. Prognostic Factors

In the univariate analysis, tumor size, total bilirubin (TBIL), albumin (ALB), ALBI-grade, AST, GGT, IL-8, PIVKA-II, and treatment with anti-PD-1 immunotherapy were significantly associated with OS. Multivariate Cox analysis demonstrated that treatment with anti-PD-1 immunotherapy was an independent prognostic factor for OS (details are presented in Table 4). In the univariate analysis, AST and receiving later-line TACE or anti-PD-1 treatment were significantly associated with TTP. However, according to multivariate Cox analysis, there were no independent prognostic factors for TTP (Supplementary Table S1).

## 4. Discussion

TACE still represents a mainstay of treatment and is often the first-line therapy in patients with advanced HCC, but TACE failure or ineligibility remains a challenge for clinicians. This study provides a later-line option for advanced HCC patients with TACE refractoriness/failure or ineligibility. In this study, all patients received TKI treatment and belonged to the TACE-failure or -ineligible advanced HCC group. The median OS and TTP were 10 months (95% CI: 5.5–14.6) and 3.5 months (95% CI: 2.3–4.7), respectively, similar to previous studies [9].

Although TKI- and anti-PD-1-based treatments are regarded as basal therapies for advanced HCC according to many international HCC guidelines, TACE is the most frequently utilized treatment [7,17]. SHARP and Oriental studies showed that sorafenib could delay advanced HCC tumor progress (median TTP, 2.8–5.5 months) and prolong patient survival (median OS, 6.5–10.7 months) [18,19]. Meanwhile, lenvatinib was not inferior to sorafenib in advanced HCC (median OS, 13.6 months vs. 12.3 months) [20]. Additionally, some studies showed that the survival time of TACE treatment was comparable to sorafenib alone [21,22]. Moreover, some reviews have demonstrated that TACE plus sorafenib/lenvatinib was more effective than sorafenib/lenvatinib or TACE alone in advanced HCC patients [23,24,25,26]. In addition, patients with diffuse HCC, portal vein tumor thrombosis, or an arterioportal/arteriovenous shunt are not suitable for TACE. Furthermore, some HCC patients became TACE-refractory after repeatedly undergoing TACE treatment. More importantly, multiple TACE treatments may cause liver function abnormalities. For these patients, switching to other TKIs or PD-1 inhibitor treatments may be a good choice, such as using sorafenib [27], lenvatinib [28], and apatinib [29,30]. However, some patients may not withstand TKI treatment, and the treatment efficacy was poor (only prolonging OS by 3–6 months). Other TKIs, for example, regorafenib [31], apatinib [32], and cabozantinib [33], may also be selected for advanced HCC. However, the treatment effects were also limited, and the ORR was 2–18.8% [34]. Although several PD-1/PD-L1 inhibitors served as second-line treatments for unresectable HCC patients with a median OS of 13.9–15.6 months [35,36,37], they are not suitable for patients with a high tumor burden and a short life expectancy of less than 3 months.

HAIC has been used classically for unresectable colorectal liver metastases [38], and is also widely performed in HCC patients. Some studies showed that HAIC with FOLFOX4 is superior to sorafenib for advanced HCC patients [39,40]. JSH consensus statements proposed that HAIC is another choice for patients with a liver function of Child–Pugh class B or worse at the time of TACE failure/refractoriness [41]. In the present study, instead of 5-FU, raltitrexed was used in the treatment of HAIC, which shortened the infusion time, improved patient comfort, and reduced hospitalization hours. A phase II prospective study showed that raltitrexed-plus-oxaliplatin-based HAIC led to a higher ORR (51.4%, 18 of 35 patients) and was considered safe and tolerable in patients with unresectable HCC [15]. Another retrospective study highlighted the efficacy of low-dose continuous HAICROX for advanced HCC patients with MPVTT, where the median survival time was 8.7 months [14]. However, the survival benefits of HAICROX treatments as a later-line therapy in TACE-TKI-failed or -refractory HCC remain unknown. In this study, we treated TACE-failed or -ineligible advanced HCC patients with HAIC with raltitrexed plus oxaliplatin. Taking the patient’s liver function and physical strength into consideration, the dosage of raltitrexed and oxaliplatin was reduced by one-third compared to the dosage in previous HAICROX studies [15]. The ORR and DCR were significantly lower in our study compared to previous results (ORR, 51.4%, 18 of 35 patients; DCR, 88.6%, 31 of 35 patients) [15], but our patients belonged to a less favorable subset of clinical baseline characteristics, such as a greater tumor burden (71.4% of patients with more than three tumors) and they were at a later BCLC stage (all of them were BCLC stage cases, 60% of patients had vascular invasion, and 54.3% of patients with extrahepatic metastasis). In fact, all of our patients were classified as TACE-failed (85.7% of patients) or TACE-unsuitable (14.3% of patients) and had received first or second lines of TKI therapy. However, our survival outcomes, including OS and TTP, were 10.0 months (95% CI: 5.5–14.6) and 3.5 months, respectively (95% CI: 2.3–4.7), comparable to those receiving HAIC with modified FOLFOX, as reported by Hsu SJ [9]. Of note, the patients in our study who received clinical benefits from HAICROX had improved OS and TTP compared to non-responders (median OS, not reached vs. 6.8 months, *p* = 0.014; median TTP, 6.5 months vs. 2.6 months, *p* = 0.023).

The results from the Check Mate 459 [42] and KEYNOTE-240 [36] trials show that anti-PD-1 immunotherapy with nivolumab or pembrolizumab as a single agent did not meet the setting primary endpoints (OS). However, in this study, anti-PD-1 treatment combination therapy was identified as an independent prognostic factor, and the patients who accepted anti-PD-1 immunotherapy and HAICROX plus TKI treatments achieved better survival outcomes; the median OS was 15.8 vs. 6.7 months (*p* = 0.01; HR = 0.329; 95% CI: 0.135–0.802) and the median TTP was 6.5 vs. 2.1 months (*p* = 0.043; HR 0.324; 95% CI: 0.113–0.926), which implied that the combination therapy may be a valuable choice and should be verified in our future studies. Consistent with another study, HAIC combined with anti-PD-1 immunotherapy (HAICAP) was superior to HAIC treatment alone for advanced hepatocellular carcinoma [43]. The potential mechanism may be that HAIC can improve anti-tumor immunity by releasing the neoplasm antigens from killed liver tumor cells [44] and increase PD-1/PD-L1 expression in the tumor microenvironment (TME). The combination of anti-PD1 therapy and locoregional therapy may also reduce the proportion of Tregs and the aggregation of myeloid-derived suppressor cells (MDSCs) [45].

Compared to previous research [14,15], HAICROX in TACE-refractory or -failed advanced HCC showed good tolerance and safety in our study. The most frequent adverse events were thrombocytopenia (17.1%), alanine aminotransferase (ALT), and aspartate aminotransferase (AST) elevation (34.29%, both), which can be managed and alleviated through dose reduction. Overall, these results show that HAICROX is a promising treatment for advanced HCC patients who failed or are unsuited to TACE combined with TKI treatments, and the subsequent HAICROX therapy prolonged the OS and TTP of advanced-stage HCC patients to some extent.

However, this study has several limitations. Firstly, this is a retrospective study, and all clinical data were obtained from a single medical center. Secondly, the categories of PD-1 inhibitors varied, which might influence the uniformity of the treatment procedure. Thirdly, our study was a single-arm study, and the sample size was small. Thus, more randomized controlled trials are required to verify the efficacy of HAICROX in this clinical setting.

In conclusion, this result highlighted that HAICROX largely prolonged the OS and TTP of advanced HCC patients who progressed on TACE-based treatment or were unsuitable/ineligible for TACE. The results provide a promising option for the later-line treatment for the majority of advanced HCC patients with TACE failure or ineligibility.

## 5. Conclusions

HAICROX showed valuable efficacy and tolerable toxicity, and it is an alternative option for advanced-stage HCC patients with TACE failure or ineligibility.

## Figures and Tables

**Table 1:** Clinical characteristics of 35 advanced HCC patients with TACE refractoriness or ineligibility.

Characteristic	*n*= 35
Age ^1^	53.0 ± 11.7
Gender	
Male	33 (94.3%)
Female	2 (5.7%)
HBV	
Negative	5 (14.3%)
Positive	30 (85.7%)
HCV	
Negative	33 (94.3%)
Positive	2 (5.7 %)
HBV + HCV coinfection	1 (2.9%)
Child–Pugh	
A	34 (97.1%)
B	1 (2.9%)
ECOG	
0	15 (42.9%)
1	20 (57.1%)
Tumor size (mm) ^1^	83.8 ± 44.8
Tumor number	
≤3	10 (28.6%)
>3	25 (71.4%)
Tumor thrombosis	
None	14 (40%)
Inferior vena cava (IVC)	7 (20%)
Main portal vein	3 (8.6%)
Branch of the portal vein	9 (25.7%)
Distant branch of portal vein	2 (5.7%)
Extrahepatic metastasis	
Yes	19 (54.3%)
No	16 (45.7%)
Number of HAIC treatments	2.3 ± 1.1
Number of previous TACE treatments	2.7 ± 2.3
Previous TKI lines	
1st	22 (62.9%)
≥2nd	13 (37.1%)
Later-line treatment	
Anti-PD-1	22 (62.9%)
TACE	10 (28.6%)
TBIL (µmol/L) ^2^	12.2 (8.5–19.2)
ALT (U/L) ^2^	36 (22–48)
AST (U/L) ^2^	44 (35–68)
Albumin (g/L) ^1^	40.3 ± 5.2
ALP (U/L) ^2^	127 (92–198.5)
GGT (U/L) ^2^	160 (78–207)
WBC (×10^9^/L) ^2^	5.76 (4.01–6.74)
Neu (×10^9^/L) ^2^	3.5 (2.1–4.6)
PLT (×10^9^/L) ^2^	161 (101–192)
L (×10^9^/L) ^2^	1.1 (0.8–1.6)
NLR ^2^	2.8 (1.8–5.1)
PLR ^2^	128.1 (91.8–151.5)
IL-6 (pg/mL) ^2^	10.4 (5.9–18.6)
IL-8 (pg/mL) ^2^	36.95 (17.75–63.73)
TNF (pg/mL) ^2^	8 (6.7–14.7)
IL-2R(U/mL) ^2^	557.5 (340–927)
ALBI (grade1/grade2/grade3)	21 (60%)/14 (40%)/0
AFP (ng/mL) ^2^	457.2 (75.3–4917)
PIVKA-II (mAU/mL) ^2^	4139 (703–28,798)

TBIL, total bilirubin; ALT, alanine aminotransferase; AST, aspartate aminotransferase; ALP, alkaline phosphatase; GGT, γ-glutamyl transpeptidase; WBC, white blood cell count; Neu, neutrophil cell count; PLT, platelet count; L, lymphocyte cell count; NLR, neutrophil–lymphocyte ratio; PLR, platelet–lymphocyte ratio; IL-6, interleukin-6; IL-8, interleukin-8; TNF, tumor necrosis factor; IL-2R, interleukin-2R; ALBI, albumin–bilirubin grade; AFP, alpha-fetoprotein; PIVKA-II, prothrombin induced by vitamin K absence II. ^1^ Data are presented as the mean ± SD. ^2^ Data are presented as the median (interquartile range).

**Table 2:** Tumor response assessed according to mRECIST after two months of HAIC treatment.

Tumor Responses	*n* = 35 (%)
CR	0
PR	4 (11.4%)
SD	16 (45.7%)
PD	15 (42.9%)
ORR	4 (11.4%)
DCR	20 (57.1%)

CR, complete response; PR, partial response; SD, stable disease; PD, progressive disease; ORR, objective response rate; DCR, disease control rate.

**Table 3:** HAICROX associated with adverse events in 35 HCC patients.

Adverse Event	Grades I *n* (%)	Grades II *n* (%)	Grades III *n* (%)	Grades IV *n* (%)
Thrombocytopenia	4 (11.4%)	2 (5.7%)	0	0
Dyspepsia/anorexia	1 (2.9%)	0	0	0
Nausea/vomiting	1 (2.9%)	0	0	0
GI bleeding	0	1 (2.9%)	0	0
Fatigue	3 (2.9%)	0	0	0
General weakness	3 (8.6%)	0	0	0
AST elevation	11 (31.4%)	1 (2.9%)	0	0
ALT elevation	11 (31.4%)	1 (2.9%)	0	0
Bilirubin elevation	3 (8.6%)	0	0	0
Hyponatremia	1 (2.9%)	0	0	0
Ascites aggravation	1 (2.9%)	0	0	0
Hepatic encephalopathy	1 (2.9%)	0	0	0

GI bleeding, gastrointestinal bleeding; AST, aspartate aminotransferase; ALT, alanine aminotransferase.

**Table 4:** Prognostic factors associated with overall survival in 35 patients (* *p* < 0.05 is considered statistically significant).

	Univariate Analysis			Multivariate Analysis		
	HR	95% CI	*p* Value	HR	95% CI	*p* Value
Gender (M/F)	0.545	0.21–1.414	0.212			
Age (<50 vs. ≥50)	0.61	0.257–1.452	0.081			
Etiology (HBC vs. HCV vs. unknown)	0.128	0.013–1.286	0.173			
TB (µmol/L)	1.066	1.014–1.121	0.007 *	1.076	0.978–1.185	0.134
Albumin (g/L)	0.879	0.800–0.966	0.013 *	1.013	0.822–1.248	0.905
ALBI-grade	2.71	1.105–6.647	0.029 *	2.982	0.259–33.046	0.385
ALT (U/L)	1.024	0.999–1.049	0.059			
AST (U/L)	1.018	1.009–1.026	<0.001 *	1.019	0.998–1.040	0.082
ALP (U/L)	1.008	1.003–1.014	0.093			
GGT (U/L)	1.004	1.001–1.008	0.027 *	0.997	0.988–1.007	0.554
WBC (×10^9^/L)	0.996	0.795–1.248	0.971			
PLT (×10^9^/L)	0.997	0.989–1.005	0.437			
Neu (×10^9^/L)	1.094	0.791–1.512	0.558			
L (×10^9^/L)	0.541	0.391–1.637	0.113			
NLR	1.094	0.853–1.403	0.48			
PLR	0.999	0.993–1.006	0.867			
IL-6	1.01	0.985–1.035	0.444			
IL-8	1.008	1.002–1.014	0.007 *	0.998	0.986–1.009	0.689
IL-2R	1.001	1–1.002	0.142			
TNF	1.004	0.996–1.013	0.284			
AFP (<400 mg/L, ≥400 mg/L)	2.242	0.924–5.439	0.741			
PIVKA-II (<400 mAU/mL, ≥400 mAU/mL)	2.286	0.668–7.828	0.001 *	0.554	0.095–3.236	0.512
Tumor size (mm)	1.01	1–1.02	0.04 *	1.01	0.992–1.028	0.28
Tumor number (1–3/>3)	1.739	0.634–4.766	0.282			
Tumor thrombosis (yes, no)	1.15	0.469–2.819	0.759			
Extrahepatic metastasis	1.2	0.508–2.837	0.677			
TACE times (1–2/>2)	1.115	0.461–2.696	0.809			
TKIs lines (1st/>2nd)	1.395	0.590–3.3	0.449			
ECOG (0/1)	0.632	0.268–1.491	0.295			
Child–Pugh class (A/B)	3.318	0.404–27.279	0.264			
Times of HAIC (1, 2/>2)	0.722	0.449–1.161	0.179			
Later-line treatment						
TACE (0, ≥1)	1.395	0.590–3.300	0.449			
Anti-PD-1 (yes, no)	0.329	0.135–0.802	0.014 *	0.267	0.075–0.953	0.042 *

TBIL, total bilirubin; ALT, alanine aminotransferase; AST, aspartate aminotransferase; ALP, alkaline phosphatase; GGT, γ-glutamyl transpeptidase; WBC, white blood cell count; Neu, neutrophil cell count; PLT, platelet count; L, lymphocyte cell count; NLR, neutrophil–lymphocyte ratio; PLR, platelet–lymphocyte ratio; IL-6, interleukin-6; IL-8, interleukin-8; TNF, tumor necrosis factor; IL-2R, interleukin-2R; ALBI, albumin–bilirubin grade; AFP, alpha-fetoprotein; PIVKA-II, prothrombin induced by vitamin K absence-II; TACE, transarterial chemoembolization; TKIs, tyrosine kinase inhibitors; ECOG, Eastern Cooperative Oncology Group; HAIC, hepatic arterial infusion chemotherapy; anti-PD-1, anti-programmed cell death protein 1 immunotherapy.

## Data Availability

The data presented in this study are available on request from the corresponding author.

## Supplementary Materials

The following supporting information can be downloaded at: https://www.mdpi.com/article/10.3390/medicina58101343/s1, Table S1: Univariate and Multivariate Analyses of Prognostic Factors for TTP of 35 patients.
